# The Role of Laboratory Markers in Primary Biliary Cholangitis: A Clinical Review and a Case Report

**DOI:** 10.3390/biomedicines14040925

**Published:** 2026-04-18

**Authors:** Raffaele Radice, Giulia Pollaroli, Michela Salvatici, Chiara Corrado, Francesca Rispoli, Stefania Pacchetti, Lorenzo Drago

**Affiliations:** 1 UOC Laboratory of Clinical Medicine with Specialized Areas, IRCCS MultiMedica Hospital, 20138 Milan, Italy; raffaele.radice@multimedica.it (R.R.); giulia.pollaroli@multimedica.it (G.P.); michela.salvatici@multimedica.it (M.S.); chiara.corrado@multimedica.it (C.C.); francesca.rispoli@biologo.onb.it (F.R.); 2 UOC Check-Up Center, IRCCS MultiMedica Hospital, 20099 Milan, Italy; stefania.pacchetti@multimedica.it; 3 Clinical Microbiology and Microbiome Laboratory, Department of Biomedical Sciences for Health, University of Milan, 20133 Milan, Italy

**Keywords:** primary biliary cholangitis, laboratory biomarkers, autoantibodies, diagnostic delay, prognostic stratification, disease monitoring

## Abstract

Background: Primary biliary cholangitis (PBC) is a rare autoimmune liver disease characterized by marked clinical and serological heterogeneity. Although diagnosis is mainly based on antimitochondrial antibodies (AMAs) and alkaline phosphatase (ALP), non-classical presentations remain a relevant cause of diagnostic delay. In this context, laboratory medicine plays a pivotal role in both diagnosis and long-term disease management. Methods: This manuscript represents a structured clinical review of laboratory biomarkers relevant to the diagnosis, monitoring, and prognostic stratification of PBC, integrated with a representative atypical case with long-term follow-up to illustrate the practical application of laboratory-driven diagnostic. Results: The analysis confirms the central role of immunological and biochemical markers in treatment monitoring and prognostic assessment, while highlighting their limitations in selected clinical scenarios. The reported case, characterized by persistent AMA negativity and consistently normal ALP levels, illustrates how expanded laboratory testing can support the identification of non-standard disease phenotypes. In this setting, parallel testing for AMA- and PBC-specific autoantibodies was essential to achieve a correct diagnosis. Moreover, alternative biomarkers, including gamma-glutamyl transferase (GGT) and selected immunological markers, provided clinically meaningful information when conventional markers were not informative. Conclusions: By integrating current evidence with a long-term clinical case, this work moves beyond a descriptive overview and proposes a practical, laboratory-driven diagnostic and follow-up framework for PBC. It highlights laboratory opportunities to facilitate timely diagnosis, appropriate prognostic stratification, and disease monitoring, including the assessment of associated comorbidities.

## 1. Introduction

Primary biliary cholangitis (PBC) is a chronic autoimmune liver disease characterized by inflammatory destruction of small- and medium-sized intrahepatic bile ducts. Although the initiating cause is not fully understood, it is believed that environmental factors, such as infections or exposure to toxic substances, in genetically predisposed individuals may lead to the aberrant expression of autoantigens on biliary epithelial cells (BECs), thereby triggering a T-cell-mediated immune response.

The disease course is generally progressive and may evolve toward hepatic fibrosis and cirrhosis, ultimately requiring liver transplantation in advanced stages. As with other forms of chronic liver disease, PBC is associated with an increased risk of hepatocellular carcinoma. From a clinical perspective, early stages are often characterized by fatigue (approximately 70%) and pruritus (up to 80%), whereas more advanced stages may present with signs of portal hypertension, including splenomegaly (30%) and jaundice (10%) [1].

From an epidemiological standpoint, PBC is considered a rare disease. According to the largest epidemiological study conducted in the United Kingdom, PBC predominantly affects women, with a female-to-male ratio of approximately 9:1. The estimated prevalence is about 35 cases per 100,000 inhabitants, with an annual incidence of 2–3 cases per 100,000 inhabitants [2,3].

Diagnosis may be challenging due to the often silent or non-specific nature of symptoms, and in this context the clinical laboratory plays a central role. After exclusion of other causes of liver disease, PBC should be suspected in the presence of persistent abnormalities in serum cholestatic markers and/or pruritus of unknown origin. According to the European Association for the Study of the Liver (EASL), a diagnosis of PBC can be established when at least two of the following criteria are met: persistent elevation (>6 months) of serum alkaline phosphatase (ALP) greater than 1.5 times the upper limit of normal (ULN), presence of antimitochondrial antibodies (AMA) at a titer ≥1:40, and histological findings compatible with PBC on liver biopsy [4,5].

AMA are detected in approximately 90–95% of patients and, due to their high specificity, represent the main diagnostic biomarker. However, a subset of patients is AMA-negative, a condition that may lead to delayed or missed diagnosis and necessitates the use of alternative immunological biomarkers [6,7]. In this setting, PBC-specific antinuclear antibodies (ANAs), directed against nuclear pore complex proteins (anti-gp210) and the soluble nuclear protein sp100 (anti-sp100), can significantly contribute to the diagnostic process. According to the current EASL recommendations, PBC can be diagnosed in patients with persistent cholestatic enzyme abnormalities (ALP and/or gamma-glutamyl transferase, GGT) in association with positivity for AMA- or PBC-specific ANAs (anti-sp100 or anti-gp210), without the need for liver biopsy [4].

In recent years, laboratory biomarkers have assumed an increasingly central role not only in the diagnosis of PBC but also in its clinical management. Biochemical and immunological parameters allow early identification of patients at risk of disease progression, assessment of response to ursodeoxycholic acid (UDCA) therapy, and risk stratification for major clinical outcomes, including the need for liver transplantation [8].

This clinical review aims to systematically map the existing evidence on laboratory biomarkers involved in the diagnosis, monitoring, and management of PBC and to illustrate their clinical relevance through a representative case report. Furthermore, based on the available scientific evidence, we seek to propose a pathology-oriented laboratory diagnostic pathway designed to ensure timely and accurate diagnosis in patients with suspected PBC.

## 2. Methods

### 2.1. Clinical Review Methodology

A comprehensive literature search was conducted using the electronic databases PubMed, Scopus, and Web of Science, covering publications from January 2000 to March 2025, considering patients with PBC as the population, laboratory biomarkers as the concept, and clinical diagnosis, disease monitoring, and prognostic assessment as the context.

A comprehensive literature search was performed using the electronic databases PubMed, Scopus, and Web of Science. The search strategy combined Medical Subject Headings (MeSH) and free-text terms related to PBC and laboratory biomarkers, including “Primary Biliary Cholangitis”, “biomarkers”, “laboratory markers”, “autoantibodies”, “alkaline phosphatase”, “AMA”, and “ANA”. In addition, the reference lists of relevant articles were manually screened to identify further eligible studies.

Studies were considered eligible for inclusion if they involved patients with PBC and evaluated laboratory biomarkers with diagnostic, prognostic, or disease-monitoring relevance. Original research articles, narrative reviews, systematic reviews, and clinical practice guidelines were included. Exclusion criteria comprised studies focusing on other cholestatic or autoimmune liver diseases, animal or in vitro studies, conference abstracts without full-text availability, and isolated case reports.

Titles and abstracts were initially screened to identify potentially relevant studies, followed by full-text review to determine final eligibility. The selected evidence was synthesized using a narrative and clinically oriented approach, with the aim of integrating classical and emerging biomarkers into a coherent laboratory-driven diagnostic and monitoring framework. No quantitative meta-analysis was performed.

### 2.2. Case Report

In addition to the scoping review, a representative clinical case was included to illustrate the application of laboratory biomarkers in routine clinical practice. Laboratory analyses for the case report were performed in a certified clinical laboratory using standardized methods. Serum biochemical parameters, including liver enzymes and cholestatic markers, were measured using automated analyzers according to the manufacturers’ instructions (Atellica^®^ Solution, Siemens Healthineers, Erlangen, Germany).

AMA and ANA were carried out using standard immunological techniques. Indirect immunofluorescence (IIF) assays were performed using an automated system (the Quanta-Lyser^®^ 3000, Inova Diagnostics, San Diego, CA, USA). ANA detection was conducted on HEp-2 cells (NOVA Lite^®^ HEp-2 slides), while AMA testing was performed on rodent tissue sections (NOVA Lite^®^, Inova Diagnostics).

Solid-phase autoantibody detection named “Liverblot” was performed using an immunoblotting technique (EUROLINE Liver Profile, EUROIMMUN, Lübeck, Germany). This assay allows in vitro qualitative and semi-quantitative detection of human IgG antibodies directed against a panel of liver-associated antigens, including gp210, AMA-M2, M2-3E (BPO), Sp100, PML, LKM-1, LC-1, SLA/LP, and Ro-52.

## 3. Results

### 3.1. Clinical Review

From a laboratory perspective, when cholestatic disease is suspected, a pathology-oriented diagnostic workflow should begin with the exclusion of viral hepatitis. Therefore, serological markers of viral hepatitis should be assessed as the first diagnostic step [4]. Once a viral etiology has been ruled out, biochemical and immunological evaluation should follow. In particular, assessment of biochemical markers of cholestasis (ALP and GGT) should be performed, followed by immunological testing for AMA- and PBC-specific autoantibodies. Requesting AMA testing in parallel with PBC-specific autoantibody assays is essential to ensure detection of atypical and AMA-negative cases [9]. Failure to include PBC-specific autoantibodies in the initial diagnostic workup may result in missed or delayed diagnosis, particularly in patients with atypical serological profiles. Accordingly, comprehensive autoantibody testing should always be performed when PBC is clinically suspected. In our reported case (see below), accurate clinical classification was achieved only through the use of an extended immunoblot assay.

Recent evidence supports the expansion of the antigenic panel used in routine diagnostics. In addition to established antigens such as gp210 and sp100, emerging biomarkers including ant-Kelch-like 12 (anti-KLHL12), anti-hexokinase 1 (anti-HK1), and anti-RPL30 antibodies have been increasingly associated with PBC [10,11]. Anti-KLHL12 antibodies are detectable in a substantial proportion of patients negative for conventional markers, being present in approximately 40% of PBC patients (42% of AMA-positive and 35% of AMA-negative cases), with a reported specificity of 96.1%. Similarly, anti-HK1 antibodies are significantly more prevalent in PBC patients than in non-PBC controls (*p* < 0.001), with a specificity of 96.9% [12]. More recently, anti-RPL30 antibodies have emerged as promising serological biomarkers, particularly in patients negative for conventional autoantibodies (AMAs, anti-gp210, anti-sp100), achieving a specificity of 100% and a sensitivity of 75%, thereby significantly improving diagnostic sensitivity [11].

**Figure 1:**
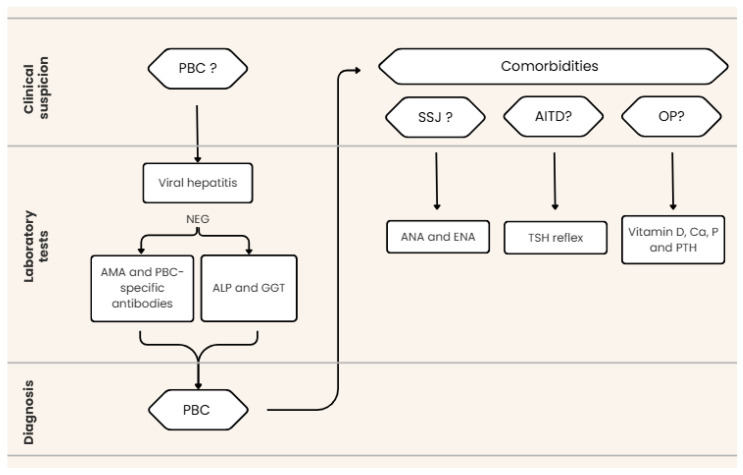
Proposed laboratory diagnostic workflow for PBC. Proposed diagnostic workflow to ensure timely and accurate diagnosis of PBC and comorbidities. The PBC-specific autoantibody panel may be expanded to include anti-gp210, anti-sp100, anti-KLHL12, anti-HK1, and anti-RPL30 antibodies.

Once a diagnosis of PBC has been established, further laboratory evaluation is warranted to assess the presence of concomitant autoimmune diseases. Additional immunological testing may provide valuable information for the identification of poly-autoimmune syndromes, given that PBC coexists with other autoimmune conditions in a substantial proportion of patients, estimated at 30–50%. Autoimmune thyroid diseases (AITD) and Sjögren’s syndrome (SSJ) are the most frequently associated conditions [9,13]. Beyond immune profiling, patients should also undergo risk assessment for metabolic bone disease (OP); evaluation of vitamin D, calcium (Ca), phosphorus (P), and parathyroid hormone (PTH) may be considered at baseline and during follow-up according to local practice and guideline recommendations [14]. Based on the available literature and the experience derived from our clinical case, a laboratory workflow capable of providing a comprehensive diagnostic response to clinicians is summarized in Figure 1. The combined request of serological tests (viral hepatitis panel), biochemical markers (GGT and ALP), and immunological assays (AMA- and PBC-specific autoantibodies) allows for the identification of PBC even in atypical cases. Subsequently, once the diagnosis has been established, comprehensive further evaluation is strongly recommended given the increased likelihood of associated comorbidities. This assessment should include screening of bone metabolism (vitamin D, Ca, P, and PTH), evaluation of thyroid function (TSH, fT3, and fT4), and screening for SSJ through autoimmune profiling (ANA and ENA). In addition, ANA testing enables the detection of anticentromere antibodies (ACA), which are recognized as prognostic markers in PBC.

Beyond their diagnostic role, PBC-associated autoantibodies also provide important insights into prognosis and disease stratification at baseline. Among these, anti-gp210 antibodies are recognized as one of the most powerful predictors of adverse outcomes [15]. Multiple studies have demonstrated that anti-gp210-positive patients have significantly poorer prognoses than antibody-negative individuals, with stronger associations with cirrhosis, hepatic functional decline, and severe cholestasis [16,17]. Similarly, anti-sp100 antibodies are more frequently detected in patients with severe PBC, and their presence may be predictive of an unfavourable disease course, irrespective of serum bilirubin levels [18,19,20]. In contrast, the relationship between AMA titers and disease severity or progression remains unclear. Although AMA titers may vary widely between patients, they tend to remain stable within individuals over time and have not been shown to reliably predict prognosis [21]. Nevertheless, qualitative aspects of AMAs, particularly the IgG3 subclass, have been strongly associated with adverse outcomes, including more advanced histological disease and higher rates of cirrhosis [22].

AMA-negative patients generally exhibit more severe clinical features and poorer long-term outcomes compared with AMA-positive patients; delayed diagnosis, as observed in the present case, may contribute to postponement of treatment initiation and, consequently, to a worse prognosis [23]. Beyond AMA status, several autoantibodies have been associated with disease progression. In particular, ACAs have been associated with a more rapid development of portal hypertension and an earlier onset of esophagogastric varices; therefore, their assessment is strongly recommended [24].

UDCA is the guideline-recommended first-line treatment for primary biliary cholangitis, acting through bile acid pool modulation, enhanced choleresis, and protection against cholestatic liver injury [25].

Assessment of response to UDCA therapy relies primarily on laboratory data, and several response criteria have been proposed based predominantly on biochemical parameters. These include ALP, bilirubin, and aspartate aminotransferase (AST) levels measured after a defined interval following treatment initiation, with additional prognostic refinement provided by markers of hepatic function such as serum albumin and platelet count (Table 1) [26,27].

Clinical studies have shown that more than half of patients achieve either a reduction in ALP of at least 40% from baseline or complete normalization within one year of UDCA therapy, supporting this biochemical response as a meaningful therapeutic target at 12 months [28,29]. In contrast, ALP and bilirubin increases were associated with worse outcomes after one year of follow-up, and ALP > 2 × ULN was associated with decreased 10 year transplant-free survival as opposed to ALP ≤ 2 × ULN (62 percent vs. 84 percent). Similarly, a bilirubin level > 1 × ULN was associated with lower 10 year transplant-free survival compared with a bilirubin level ≤ 1 × ULN (41 percent vs. 86 percent) [30].

In selected clinical scenarios where ALP levels remain within the reference range, as observed in our case, alternative biomarkers may be required to support diagnosis and disease assessment. GGT is emerging as an independent prognostic marker. Persistent elevation of GGT despite therapy has been associated with unfavourable outcomes, independently of ALP levels. This implies that patients with well-controlled ALP, but persistently elevated GGT may still be at increased risk. In such cases, careful evaluation of persistently elevated GGT is advisable, but rather investigating potential concomitant causes (e.g., metabolic-associated fatty liver disease or drug-induced liver injury) and considering treatment intensification once alternative explanations have been excluded. Conversely, a marked reduction in GGT during therapy represents a favorable prognostic sign; a decrease of approximately ≥34% from baseline has been associated with improved long-term survival [31].

Elevated levels of immunoglobulin M (IgM) are commonly found in PBC approximately 80% of patients [32,33]. Although IgM is not included in the assessment formula, studies have shown that IgM improves with treatment of PBC with UDCA [34]. Thus, IgM may be of clinical relevance in patient populations with diagnosed PBC and can be used to monitor response to therapy [35].

With regard to treatment response, recent studies have reported that UDCA therapy in responding patients may lead to a reduction in AMA titers, and that some patients may even lose positivity for anti-gp210 antibodies during treatment. However, the clinical and prognostic significance of these serological changes requires further validation [36,37].

In addition to traditional clinical and biochemical markers, gut microbiome analysis is gaining increasing attention in PBC. A growing body of evidence indicates that intestinal dysbiosis is common in PBC patients [38]. Compared with healthy controls, patients with PBC show a reduced abundance of beneficial bacterial taxa (such as *Bacteroidetes*) and an increased prevalence of opportunistic pathogens, including members of *Firmicutes* and *Proteobacteria* [39]. Gut microbiota dysbiosis is thought to contribute to disease progression in PBC [40]. Moreover, specific microbial phyla, such as *Actinobacteriota*, *Desulfobacterota*, and *Verrucomicrobiota*, appear to influence the therapeutic response to UDCA, making them promising candidate biomarkers for treatment stratification and personalized therapeutic approaches [41]. This emerging evidence in microbiology opens new perspectives not only for diagnostic refinement but also for predicting and monitoring response to therapy, even if large-scale and long-term clinical studies are expected to promote the broad application of gut microbiota in the clinic [42].

### 3.2. Case Report

The patient was a male born in 1965 who underwent long-term clinical and laboratory follow-up beginning in 2012, when he first sought medical attention for the onset of ocular dryness. Family history revealed a predisposition to intestinal neoplastic disease, as the patient’s father had previously undergone surgery for colon carcinoma. This finding prompted the patient to adhere to regular clinical surveillance. He reported never having smoked, occasional alcohol consumption (approximately two glasses of wine per week), and an overall healthy lifestyle.

Initial laboratory investigations performed in 2012 revealed mild hypercholesterolemia, elevated gamma-glutamyl transferase (GGT: 144 U/L; reference range 6–40 U/L), increased alanine aminotransferase (ALT: 73 U/L; reference range 8–41 U/L), negative viral serology, normal bilirubin levels (0.57 mg/dL; reference range 0.1–1.2 mg/dL), and ferritin within the reference range (270 ng/mL; reference range 10–291 ng/mL). Given the presence of ocular dryness, a possible association with SSJ was hypothesized, and an immunological screening was initiated. ANA and AMA were both negative, as was the ENA panel, including anti-Sm, anti-RNP, anti-SSA, anti-SSB, and anti-Scl-70 antibodies.

Abdominal ultrasound examination demonstrated early hepatic steatosis, which was attributed to a possible metabolic etiology. At this stage, autoimmune liver disease was excluded, and the patient was advised to follow a hypocaloric, low-carbohydrate diet to improve the lipid profile.

During follow-up in 2013, ANA positivity was detected for the first time, with a homogeneous pattern at a titer of 1:80, in association with persistently abnormal liver biochemistry, characterized by elevated GGT (133 U/L) and ALT (50 U/L). In 2015, in addition to the persistent alteration of liver enzymes, a significant increase in ANA titer (1:160), again reported as a homogeneous pattern, was observed.

**Figure 2:**
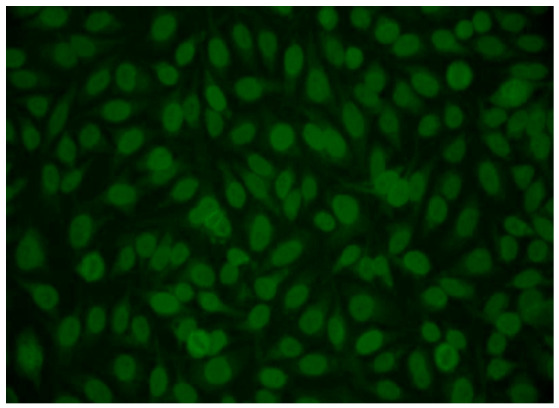
ANA pattern shows rim punctate nuclear envelope (AC-12). Magnification 40×.

In 2019, the patient reported progressive weight loss and sleep disturbances. In this context, ANA indirect immunofluorescence revealed a homogeneous pattern associated with enhanced fluorescence at the nuclear envelope, described as a “peripheral” pattern. A diagnostic turning point occurred in 2024, when the adoption of the updated nomenclature proposed by the International Consensus on ANA Patterns (ICAP) allowed accurate classification of the ANA pattern as nuclear envelope speckled, AC-12 (Figure 2), which is highly suggestive of the presence of anti-gp210 antibodies [43].

Based on these findings, the clinical pathologist recommended targeted testing, which confirmed marked positivity for anti-gp210 antibodies. In the presence of persistent cholestatic enzyme abnormalities, particularly elevated GGT, and anti-gp210 positivity, a diagnosis of primary biliary cholangitis was established [4]. In 2024, noninvasive assessment of fibrosis was performed using transient elastography (FibroScan). Liver stiffness measurement was 4.5 kPa (IQR 0.5 kPa), indicating the absence of significant fibrosis (range F0–F1). The IQR/median ratio was well below 30%, confirming the high reliability of the measurement. The controlled attenuation parameter (CAP) was 287 dB/m, suggesting moderate hepatic steatosis.

No clinical, laboratory, or imaging evidence of fibrosis progression was documented during follow-up. Following diagnosis, first-line therapy with ursodeoxycholic acid (UDCA) was initiated at a dose of 600 mg/day (300 mg twice daily).

**Figure 3:**
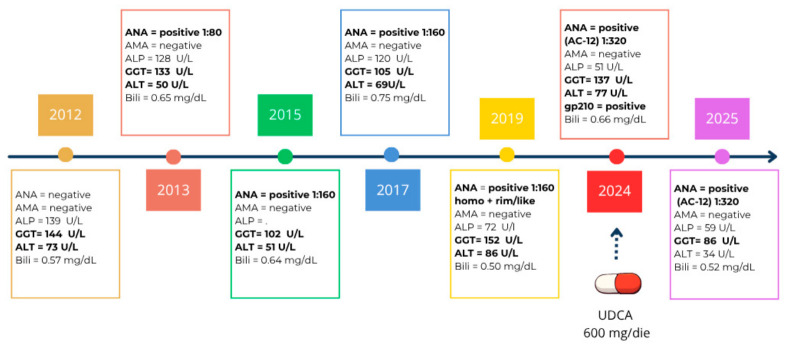
Case report timeline shows change in laboratory biomarker during follow-up. Bold values indicate results above the normal range.

In 2025, this treatment resulted in a reduction of GGT levels to 86 U/L, representing a marked decrease compared with values observed over the previous thirteen years and consistent with an adequate biochemical response to therapy [31]. Figure 3 provides a graphical summary of the changes in the main laboratory biomarkers over the 13-year follow-up period.

## 4. Discussion

The aim of this clinical review was to critically synthesize the available evidence on laboratory biomarkers involved in the diagnosis, monitoring, and prognostic assessment of PBC. In addition, a representative clinical case was included to illustrate the application of laboratory biomarkers in real-world clinical practice. The research question guiding this review was “Which laboratory markers have been investigated for the diagnosis, disease monitoring, and prognostic stratification of PBC, and what is the role of emerging biomarkers?”

Within this framework, the present study reflects the heterogeneous clinical and serological spectrum of PBC and underscores the pivotal role of advances in laboratory medicine in disease identification and management. Given the broad diagnostic capabilities offered by modern laboratory testing, complex cases such as the one described may substantially benefit from strengthened communication between laboratory specialists and clinicians. In this instance, the diagnostic turning point followed recognition of the nuclear envelope pattern (AC-12) and the subsequent recommendation to test for anti-gp210 antibodies, ultimately allowing accurate disease classification.

In the future, harmonization of interpretative reporting for highly specific ANA patterns may prove valuable. For example, standardized comments such as “The presence of an AC-12 pattern is highly suggestive of anti-gp210 antibodies; targeted testing is recommended” could improve diagnostic precision. A similar approach applies to the detection of the multiple nuclear dots pattern (AC-6), which is strongly suggestive of anti-sp100 antibodies, also included among the diagnostic markers for PBC. In such cases, interpretative comments could meaningfully support non-specialist clinicians in initiating a PBC-oriented diagnostic pathway. In this context, ICAP is, and will continue to be, fundamental in standardizing ANA pattern classification and improving communication regarding associated antibody specificities.

Furthermore, as previously demonstrated by Gaiani et al. and illustrated in Figure 3, when clinical suspicion is high, it may be appropriate to combine AMA testing with a solid-phase screening assay (immunoblot or ELISA) including the principal PBC-specific antigens (gp210 and sp100). In selected cases, this parallel testing strategy may represent the decisive element for establishing a correct diagnosis and has been shown to significantly improve diagnostic sensitivity [9].

Novel autoantibodies, including anti-KLHL12, anti-HK1, and anti-RPL30, demonstrate promising diagnostic potential and may eventually be incorporated into PBC-specific autoantibody panels; however, further validation studies are required before routine implementation.

Once a diagnosis of PBC has been established, investigation of commonly associated comorbidities is recommended, particularly AITD and, most importantly, SSJ, for which anti-SSA and anti-SSB antibodies represent key serological markers [44].

Beyond specialized autoimmune testing, clinical chemistry provides several relevant biomarkers, particularly among cholestatic indices. Growing evidence suggests that reliance exclusively on ALP may be reductive. GGT has acquired a more established complementary role, while further research is needed to clarify the independent prognostic contribution of bilirubin in earlier disease stages.

Selection of biomarkers for longitudinal follow-up should primarily be guided by baseline abnormalities, and secondarily by the strength of available scientific evidence. Table 2 provides a practical overview of currently available biomarkers and offers a semi-quantitative assessment of their prognostic relevance. In the present case, the absence of abnormalities in bilirubin and ALP limited their usefulness for monitoring; in this context, GGT served as an alternative biomarker, providing additional insight into biochemical response and potential disease activity. Recent studies have increasingly highlighted the relevance of GGT in follow-up assessment.

Similarly, accumulating evidence suggests that gut microbiota profiles and qualitative immunological characteristics, such as IgG subclass distribution, may provide additional insights into disease heterogeneity. Although these markers are not yet suitable for routine clinical application, they represent promising candidates for future translational and clinical research.

## 5. Conclusions

In conclusion, this clinical review and representative case analysis highlight the central role of integrated laboratory assessment in the diagnosis and longitudinal management of PBC, while emphasizing practical strategies to reduce diagnostic delay, particularly in atypical clinical scenarios.

Expert interpretation of ANA patterns, together with clear and structured reporting that includes recommendations for targeted follow-up testing, represents a fundamental diagnostic tool. In the future, carefully designed reflex testing strategies may be evaluated to allow laboratories to autonomously extend serological investigations when highly suggestive patterns are identified.

In cases of strong clinical suspicion, parallel testing with solid-phase assays, such as an immunoblot or ELISA incorporating PBC-specific antigens, may serve as a valuable complementary approach to indirect immunofluorescence, particularly for detecting AMA-negative presentations.

With regard to longitudinal monitoring, follow-up strategies should primarily be guided by baseline biochemical abnormalities and by selecting the most informative and dynamically responsive biomarker in the individual patient (ALP or GGT). Personalized interpretation of laboratory trends may therefore enhance monitoring precision.

The proposed laboratory-oriented diagnostic workflow and follow-up utility score are intended as structured tools to support clinical reasoning and biomarker prioritization rather than as prescriptive clinical guidelines.

## 6. Limitations and Future Research

This study has several limitations. First, it represents a structured clinical review rather than a formal systematic evidence synthesis; therefore, although major and clinically relevant studies were considered, quantitative meta-analytic assessment was not performed. Second, the case report reflects a single patient and cannot be generalized to the broader PBC population. Finally, the proposed laboratory-oriented diagnostic framework has not been externally validated and should not be interpreted as a formal clinical guideline. Future research should focus on prospective validation of integrative biomarker algorithms, clarification of the independent prognostic value of emerging autoantibodies, evaluation of composite biochemical models beyond isolated ALP thresholds and standardization of translational biomarkers, including immunological profiling and microbiome-related signatures.

## Figures and Tables

**Table 1:** Main biochemical response criteria to UDCA therapy in PBC. Summary of the principal biochemical response models to ursodeoxycholic acid (UDCA) therapy in PBC, including classical criteria (Paris I/II, Toronto), continuous risk scores (GLOBE, UK-PBC), and the recently proposed Xi’an criterion for early treatment assessment. For each model, thresholds, timing of evaluation, and clinical utility in predicting prognosis or guiding treatment decisions are reported.

Criterion	Formula	Timepoint	Prognostic Significance
Paris I	ALP ≤ 3 × ULN (plus AST ≤ 2 × ULN and normal bilirubin)	12 months	Identifies high-risk patients; nonresponders (failing criteria) had ~2.5× higher death/transplant risk (10 yr survival ≈ 50%).
Paris II	ALP ≤ 1.5 × ULN (AST ≤ 1.5 × ULN, normal bilirubin)	12 months	Tailored to early-stage PBC; responders (meeting criteria) showed no progression (100% 5–15 yr survival), nonresponders fared worse.
Toronto	ALP < 1.67 × ULN (~184 IU/L)	24 months	Predicts histologic progression; >80% of “nonresponders” (ALP ≥ 1.67×) developed fibrosis progression by 10 yr (OR ≈ 12).
GLOBE Score	Multivariable score (age, ALP, bilirubin, albumin, platelets at 12 mo)	12 months	Continuous risk score for transplant-free survival; score > 0.30 (≈40% of pts) confers HR ~5.5 for death/transplant vs. general population.
UK-PBC Score	Multivariable score (albumin, platelets, bilirubin, AST/ALT, ALP at 12 mo)	12 months	Predicts 5/10/15 yr risk of end-stage liver disease with high accuracy (AUROC ≈ 0.95–0.96); identifies highest-risk patients needing additional therapy.
Xi’an	ALP ≤ 2.5 × ULN (AST ≤ 2 × ULN, TBIL ≤ ULN)	1 month	Early-response criterion: responders had 97% 5-yr event-free survival vs. 64% in nonresponders. Outperforms other models in flagging rapid progressors.

**Table 2:** Laboratory biomarkers for follow-up in PBC. The table summarizes established and emerging laboratory biomarkers according to their clinical role and relative utility in patient follow-up. A semi-quantitative follow-up utility score (0–3) is provided to reflect the strength of evidence supporting each marker in longitudinal disease monitoring and prognostic assessment. Higher scores indicate greater relevance for routine follow-up, whereas lower scores identify markers with limited or currently investigational clinical applicability.

Marker	Clinical Role in Follow-Up	Follow-Up Utility Score (0–3)	Key Notes
ALP	Primary marker for treatment response and prognosis	3	Core component of all UDCA response criteria; ALP > 2× ULN linked to reduced transplant-free survival
Bilirubin	Marker of advanced disease and survival	3	Strongest predictor of mortality and transplant risk
Albumin	Hepatic synthetic function	2	Reflects advanced disease and prognosis
Platelet count	Hepatic synthetic function	2	Decline suggests cirrhosis progression
GGT	Independent prognostic and monitoring marker	2	Persistent elevation predicts poorer outcomes despite normal ALP
AMA IgG3 subclass	Prognostic refinement	2	Associated with advanced histology
AST	Ancillary marker in response criteria	1	Limited specificity when used alone
IgM	Ancillary disease activity marker	1	Improves with UDCA; not diagnostic
Loss of AMA/anti-gp210	Experimental monitoring marker	0–1	Prognostic significance not yet validated
Gut microbiota profiles	Emerging prognostic tool	0	Not ready for clinical follow-up

## Data Availability

No new data were created or analyzed in this study.
